# Supplementary material for: Tead1a Initiates Transcriptional Priming Through the TEAD1a/YAP‐Notch1‐Spi1/Cebpα Axis to Promote Neutrophil Fate
Source: Adv Sci (Weinh). 2025 Aug 28;12(41):e05441. doi: 10.1002/advs.202505441 (PMC12591127; doi:10.1002/advs.202505441)
Supplement: Supplementary file 4 — Supplementary Dataset [file ADVS-12-e05441-s004.zip › 3-4.zip!/3-4/02-4.cluster/cluster10/Enrich/ego_BP.pdf]

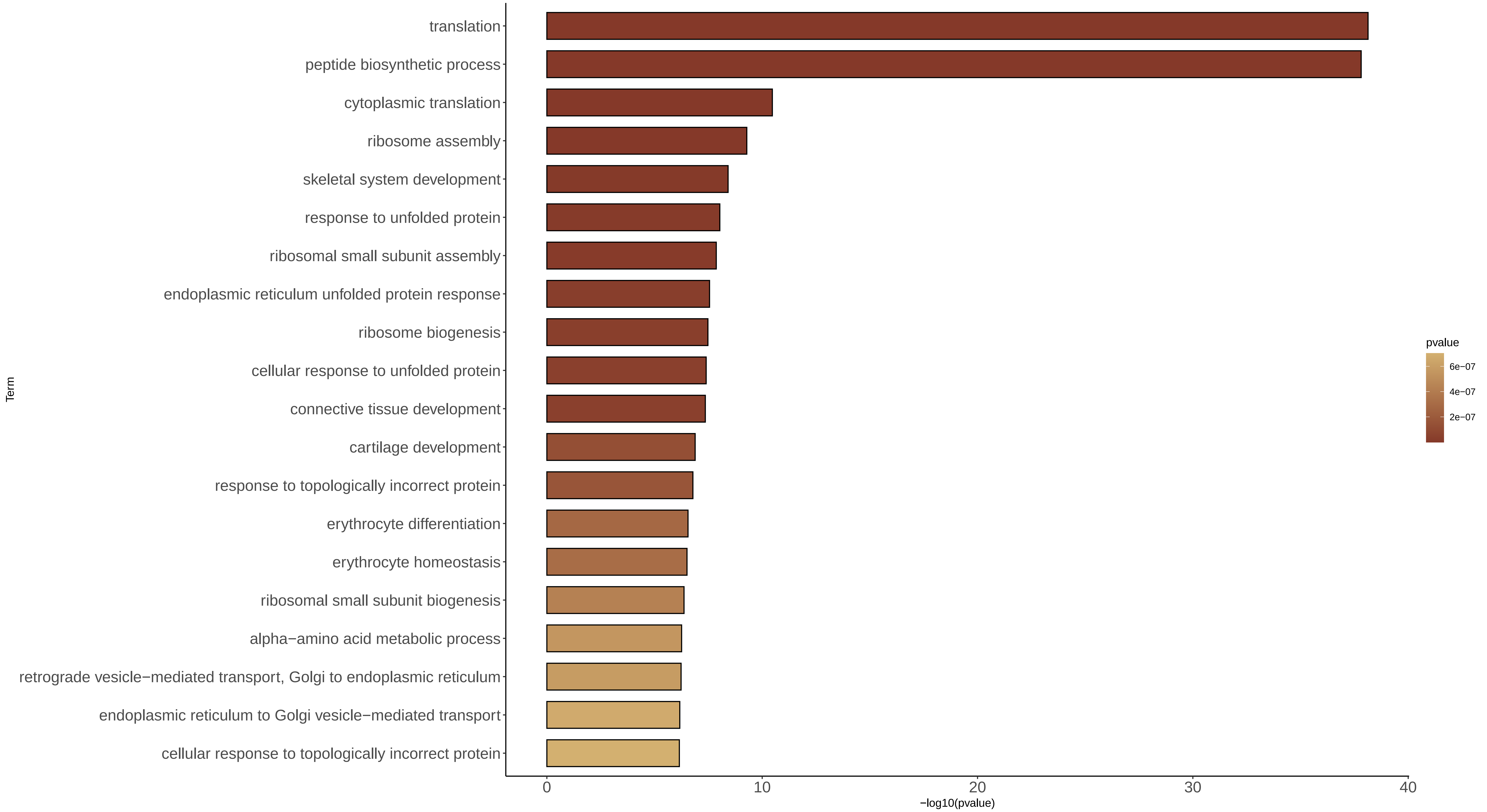
translation
peptide biosynthetic process
cytoplasmic translation
ribosome assembly
skeletal system development
response to unfolded protein
ribosomal small subunit assembly
endoplasmic reticulum unfolded protein response
ribosome biogenesis
cellular response to unfolded protein
connective tissue development
cartilage development
response to topologically incorrect protein
erythrocyte differentiation
erythrocyte homeostasis
ribosomal small subunit biogenesis
alpha−amino acid metabolic process
retrograde vesicle−mediated transport, Golgi to endoplasmic reticulum
endoplasmic reticulum to Golgi vesicle−mediated transport
cellular response to topologically incorrect protein
Term
0
10
20
30
40
−log10(pvalue)
pvalue
6e−07
4e−07
2e−07
